# The Evaluation of Left Ventricle Ischemic Extent in Patients with Significantly Suspicious Cardiovascular Disease by ^99m^Tc-Sestamibi Dynamic SPECT/CT and Myocardial Perfusion Imaging: A Head-to-Head Comparison

**DOI:** 10.3390/diagnostics11061101

**Published:** 2021-06-16

**Authors:** Hung-Pin Chan, Chin-Chuan Chang, Chin Hu, Wen-Hwa Wang, Nan-Jing Peng, Yu-Chang Tyan, Ming-Hui Yang

**Affiliations:** 1 Department of Nuclear Medicine, Kaohsiung Veterans General Hospital, Kaohsiung 813, Taiwan; markscience05@hotmail.com (H.-P.C.); ghu@vghks.gov.tw (C.H.); 2 Department of Nuclear Medicine, Kaohsiung Medical University Hospital, Kaohsiung 807, Taiwan; chinuan@kmu.edu.tw; 3 School of Medicine, Kaohsiung Medical University, Kaohsiung 807, Taiwan; 4 Department of Electrical Engineering, I-Shou University, Kaohsiung 840, Taiwan; 5 Department of Internal Medicine, Division of Cardiology, Kaohsiung Veterans General Hospital, Kaohsiung 813, Taiwan; whwang@vghks.gov.tw; 6 Department of Nuclear Medicine, Taipei Veterans General Hospital, Taipei 112, Taiwan; nanjingpeng@gmail.com; 7 National Yang-Ming University, School of Medicine, Taipei 112, Taiwan; 8 Research Center for Environmental Medicine, Department of Medical Imaging and Radiological Sciences, Kaohsiung Medical University, Kaohsiung 807, Taiwan; 9 Graduate Institute of Animal Vaccine Technology, National Pingtung University of Science and Technology, Pingtung 900, Taiwan; 10 Institute of Medical Science and Technology, National Sun Yat-sen University, Kaohsiung 804, Taiwan; 11 Department of Medical Research, Kaohsiung Medical University Hospital, Kaohsiung 807, Taiwan; 12 Department of Medical Education and Research, Kaohsiung Veterans General Hospital, Kaohsiung 813, Taiwan; 13 Center of General Education, Shu-Zen Junior College of Medicine and Management, Kaohsiung 821, Taiwan

**Keywords:** CAD, Dynamic SPECT/CT, myocardial perfusion imaging, LV ischemic extent

## Abstract

Heart disease is the second most common cause of mortality in Taiwan, mainly coronary artery disease (CAD).Quantitative coronary blood flow has been collected by dynamic single-photon emission computed tomography (Dynamic SPECT/CT) for CAD diagnosis in previous studies. However, few studies defined the extent of left ventricle (LV) ischemia on Dynamic SPECT/CT for predicting significant coronary artery stenosis. This study evaluates the extent of LV ischemic blockage in patients suspected of CAD who were referred by cardiologists. A total of 181 patients with suspected CAD were enrolled. They underwent ^99m^Tc-Sestamibi (MIBI) Dynamic SPECT/CT survey before cardiac intervention. Dynamic SPECT/CT has better sensitivity (88%), specificity (96%), and accuracy (94%) compared with those of semi-quantitative MIBI MPI (more than 10%). Results indicated that5% of the LV ischemic extent can yield positive PCI results (>70% stenosis in coronary arteries) compared with the moderate abnormal extent of at least 15% of LV. When the percentage of combined moderate abnormal extent and ischemia extent of LV reaches 27.3%, positive PCI results may be indicated. This study revealed Dynamic SPECT/CT has greater sensitivity, specificity, and accuracy as compared with MPI. Thus, the severity of abnormal perfusion extent of LV on Dynamic SPECT/CT might be beneficial to predict positive PCI results in patients with significant suspicion CAD.

## 1. Introduction

Heart disease is the second most common cause of mortality in Taiwan, mainly coronary artery disease (CAD). The main cause of CAD is atherosclerosis, in which unstable plaques are formed in coronary arteries and may cause coronary stenosis, myocardial ischemia, acute myocardial infarction, and even death [1,2]. Early detection of CAD is an important issue for clinical practice. In nuclear medicine, pharmacologic stress with Dipyridamole (vasodilator) is frequently used for the noninvasive detection of CAD with radionuclide myocardial perfusion imaging (MPI). However, MPI has some limitations in clinical use, for example, a diagnosis of balanced ischemia, post-myocardial infarction, or cardiomyopathy may cause interference or misdiagnosis [3,4,5]. There are some parameters for CAD diagnosis using cardiac positron emission tomography (PET), including myocardial blood flow (MBF), coronary flow reserve (CFR), and coronary flow capacity (CFC) [6]. CFR is the ratio of stress blood flow during coronary vasodilation to resting blood flow that indicates the assessment of coronary vasodilator capacity and clinical outcome; coronary flow capacity (CFC) is considered to determine the severity of coronary disease by integrating stress MBF, rest MBF, and CFR of cardiac PET/CT [6]. This kind of parameter cannot be offered by MPI for CAD diagnosis. Quantitative assessment of MBF by PET also has better diagnostic accuracy and shows better spatial resolution and accurate attenuation correction [7]. It is also associated with a high major adverse cardiac event and risk of mortality that can be improved after revascularization [8,9]. Cyclotrons are expensive medical instruments and are not equipped in every hospital. Since most cardiac PET/CT radiotracers are produced by cyclotron, cardiac PET imaging is limited. Previously, Hsu et al. presented the use of dynamic single-photon emission computed tomography/computed tomography (Dynamic SPECT/CT) to measure absolute MBF and CFR to enhance CAD detection with increased diagnostic benefits [10]. There are only a few studies that have concluded significant left ventricle (LV) ischemic extent in cardiologist referred CAD patients on Dynamic SPECT/CT before catheterization for prior cardiologist consultation. The goal of this study was to investigate the correlation of LV ischemic extent of the entire myocardium by Dynamic SPECT/CT with CAD patients.

## 2. Materials and Methods

### 2.1. Patients

**Figure 1:**
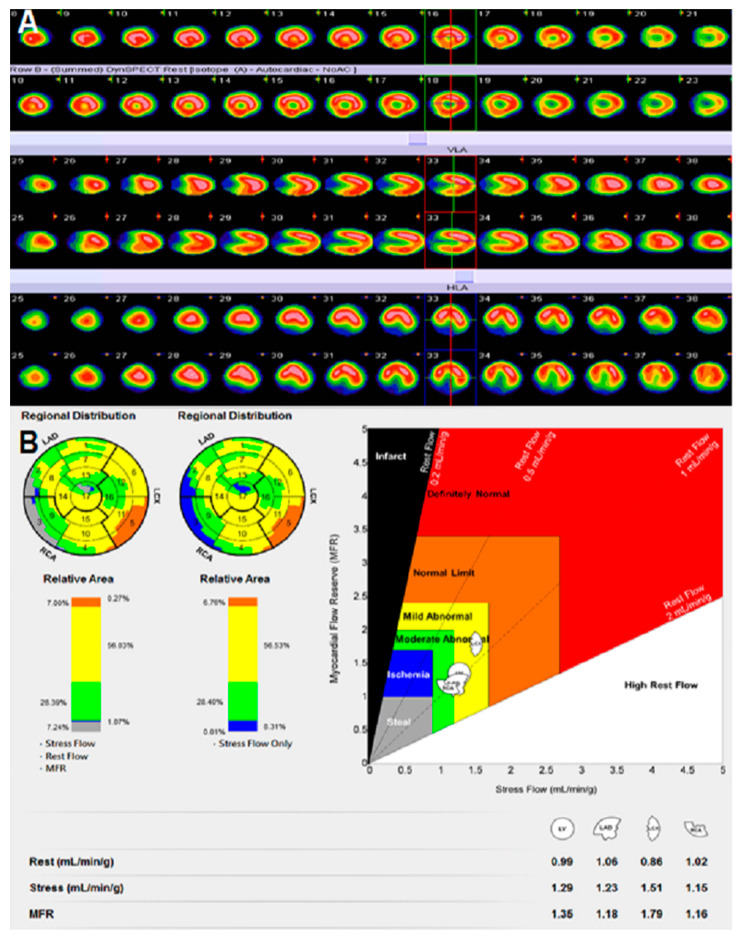
A 62 y/o male patient complained about chest pain and asked for help atthe CV OPD. He suffered from dyslipidemia, a risk factor for cardiovascular disease. He was referred for Dynamic SPECT/CT examination. Semi-quantitative MPI had no abnormal perfusion defect seen on (**A**). Quantitative coronary flow data of Dynamic SPECT/CT and CFC is shown on (**B**). CFC revealed moderate abnormal perfusion in RCA territory. Ischemic extent of LV was measured 8.3 (blue color), moderate abnormal perfusion of LV was measured as 28.4% (green color), and mild abnormal perfusion of LV was measured as 56% (yellow color). One month later, cardiac catheterization was arranged for him. Results of PCI were noted LCX-M 55% stenosis and RCA-orifice 70% stenosis. Due to this, the patient underwent PCI with stents over RCA territory. After PCI intervention, patient’s symptoms were improved and dyslipidemia was controlled with medication.

A total of 181 patients who were suspected of having CAD and were referred by cardiologists to our nuclear medicine department for Dynamic SPECT/CT examination. Patients presented typical cardiac symptoms, including chest tightness, chest pain, dyspnea on exertion or palpitation, and had visited the cardiovascular outpatient department (CV OPD) between January 2016 and December 2017. Some diseases or factors may affect the accuracy, including certain cardiomyopathy, significant congenital heart disease, heart failure, certain pericarditis, hypersensitivity to caffeine, or ever having had side effects after caffeine intake. Patients who had suffered from any of these diseases or factors were excluded from this study. Cardiac catheterization was arranged within three months after Dynamic SPECT/CT examination (Figure 1). However, 14 patients of our study were excluded for data evaluation, including three patients who had caffeine intake <24 h before study; 10 patients who did not have PCI for personal reasons; one patient had PCI at another hospital. Underlying diseases of patients included hypertension (HTN), diabetes (DM), or hyperlipidemia which was collected from patient clinical notes. The characteristics of the enrolled patients are given in Table 1. The study design was approved by the Institutional Review Board, Kaohsiung Veterans General Hospital (17-CT10-12).

➢ Excluded caffeine intake before study—three patients.
➢ Excluded not done PCI—10 patients
➢ Excluded PCI done at other hospital—one patient
➢ There were 167 patients for data analysis.

### 2.2. Dynamic SPECT/CT Imaging

All patients were instructed to discontinue medication and to abstain from caffeinated beverages and cigarettes for at least 72 h before Dynamic SPECT/CT studies. Patients underwentrest/dipyridamole-stress ^99m^Tc-Sestamibi (MIBI) on a one-day protocol that was performed on a dedicated Siemens Symbia-T2 SPECT system. At rest, a 13 mCi MIBI injection was followed by low dose CT for attenuation correlation. Three hours later, the stress agent, dipyridamole, was infused intravenously at a dose of 0.56 mg/kg. Another second dose of 30 mCi MIBI injection was given after terminating the dipyridamole infusion. Quantitative coronary blood flow and coronary flow reserve were analyzed by MyoFloQ software [10]. Semi-quantitative MIBI myocardial perfusion imaging (MPI) was also analyzed.

### 2.3. Image Evaluation

Dynamic SPECT/CT images were reconstructed with 3D-OSEM, physical correction, and noise filtration with the matched projection method [11]. To generate time–activity curves (TAC) for the blood pool and myocardium, regions of interest (ROI) were drawn for blood pooling and myocardial activity. A series of sectorial regions are placed automatically at the endocardial border of the short-axis image planes [12,13]. Dynamic datasets were analyzed using radial activity. TAC was fitted to get estimated MBF values with a three-compartment model developed by Hutchins et al. [14,15]. Coronary flow reserve (CFR) was defined as the ratio of hyperemic MBF (by dipyridamole injection) to resting MBF [16].MPI ischemia was determined by visual assessment (including reversible defect or persistent defect) and was defined ≤8% ischemic extent of LV on Dynamic SPECT/CT. This is according to a prior study [8]. Three experienced nuclear medicine physicians (H.P. Chan, C. Hu, and N.J. Peng) who have at least 10 years of experience in interpreting SPECT/CT or PET images and who were blind to patient clinical presentation, analyzed data of this study. For LV ischemic extent prediction, Dynamic SPECT/CT results were separated into three groups according to the severity of abnormal perfusion extent in the left ventricle, which includes ischemic perfusion, moderate abnormal perfusion, or coexistence of ischemic and moderate abnormal perfusion. We next sought to determine the cut-off of every perfusion for optimal prediction of the significant stenosis of the coronary artery (>70% stenosis) in PCI.

### 2.4. Cardiac Catheterization

Cardiac catheterization not only is an invasive procedure but is also one of the gold standards for CAD diagnosis and percutaneous coronary intervention treatment. The procedure to insert a sterile catheter into the coronary artery of the heart was performed by puncture of the femoral artery over the groin region, or the radial artery over the wrist region. We defined and selected one or more coronary arteries with a diameter more than 70% stenosis for positive PCI results. Patients who had at least one coronary artery stenosis would undergo cardiac stent treatment.

### 2.5. Statistical Analysis

Results are shown as mean±standard deviation or frequencies (%). Statistical significance of the association of positive and negative Dynamic SPECT/CT was assessed, including stress MBF, rest MBF, and CFR by using t test. The differences of sensitivity, specificity, or accuracy between Dynamic SPECT/CT and MPI were done with McNemar’s test and a two-sample test of proportions for positive and negative predictive values. The 95% confidence intervals for all diagnostic efficacy measures were derived at the 100(1-α)% confidence interval. In exploratory analysis, the receiver operating characteristic (ROC) curve was used to assess the best ischemic extent, moderate abnormal perfusion extent, or combined ischemic and moderate abnormal extent of the left ventricle for the positive findings in cardiac catheterization. A *p* value of < 0.05 was considered statistically significant. All statistical analyses were performed using the SPSS software package (SPSS, version 17.0).

## 3. Results

### 3.1. Overall Population

A total of 181 patients presented typical cardiac symptoms that showed significant suspicion of CAD by cardiologist evaluation and were referred to our department for Dynamic SPECT/CT examination. Of those, three patients were excluded due to reported caffeine intake <24 h before the study (to avoid examination interference). PCI was not done for 10 patients for personal reasons; for example, medical treatment was wanted first, or hesitation for PCI. It is worth mentioning that caffeine intake decreased MBF and CFR in healthy volunteers according to previous PET studies and it yielded false positive results of PET/CT [17,18], due to caffeine causing inadequate hyperemia of the coronary artery. In our study, patients had been requested to abstain from caffeinated beverages and cigarettes at least 72 h before the study to avoid false positives. In our population, the mean age of patients is 68.5 ± 7.2 years old. There are 99 males (55%) and 82 females (45%) in our study. Thirty-two out of 181 patients (17%) were smokers or ex-smokers. Patients were noted to be at cardiac risk, including HTN (85%), DM (62%), or dyslipidemia (50%) collected from clinical notes. 

### 3.2. The Analysis of Semi-Quantitative MPI and Quantitative Dynamic SPECT/CT

Among the 167 patients examined, the relationship of semi-quantitative and quantitative data was as follows: both presented ischemia on MPI and Dynamic SPECT/CT in 26 patients (15.5%); normal MPI, but ischemia on Dynamic SPECT/CT in 16 patients (9.5%); ischemia on MPI, but normal Dynamic SPECT/CT in 27 patients (16.1%); both normal on MPI and Dynamic SPECT/CT 98 patients (58.6%). It revealed that over fifty percent of our patients had negative results of both imaging modalities. MPI presented more positive findings than Dynamic SPECT/CT in patients. 

### 3.3. The Relationship of Semi-Quantitative MPI with Cardiac Catheterization

The relationship of semi-quantitative MPI results and PCI are given in Table 2. The patients were diagnosed with CAD by one or more coronary arteries with a diameter of more than 70% stenosis on PCI. Twenty-six patients (16%) revealed abnormal MPI results, but coronary arteries of <70% stenosis were seen on PCI. A total of 15 patients (9%) showed no ischemic MPI, but positive results of PCI. A total of 27 patients (16%) revealed both positive MPI and PCI results. In our study, MPI had sensitivity (64.3%; 95% confidence interval (CI), 49–78%), specificity (79.2%; 95% CI, 72–86%), accuracy (75%), positive predictive value (PPV) (50.1%; 95% CI, 37–64%), and negative predictive value (NPV) (86.8%; 95% CI, 80–93%). Of the 181 patients, 99 (>50%) had a negative MPI result, coexisting with a negative PCI result.

### 3.4. The Relationship of Dynamic SPECT/CT with Cardiac Catheterization

The two procedures are analyzed in Table 3. The average rest blood flow of LV was calculated to be 1.04 ± 0.2 mL/min/g in normal group and 1.1 ± 0.2 mL/min/g in CAD group. There appeared to be no obvious difference between normal and CAD patients. MBF (2.2 ± 0.8 vs. 1.5 ± 0.3) and CFR (2.6 ± 0.8 vs. 1.4 ± 0.4) were predominantly decreased in the CAD group, compared with the normal group (*p*<0.01; Table 4). Only five patients (3%) revealed abnormal Dynamic SPECT/CT but a normal PCI result. Additionally, only five patients (3%) showed normal Dynamic SPECT/CT but positive PCI. A total of 37 patients (22%) revealed both positive MPI and PCI results. In this study, Dynamic SPECT/CT had sensitivity (88%; 95% CI, 78–97%), specificity (96%; 95% CI, 92–99%), accuracy (94%), PPV (92.5%; 95% CI, 78–97%), and NPV (96%; 95% CI, 92–99%). As compared with semi-quantitative MPI and quantitative Dynamic SPECT/CT, we found Dynamic SPECT/CT to be more than 10% in sensitivity, specificity, and accuracy. More true normal patients were noted in Dynamic SPECT/CT (120 patients) than MPI (99 patients). The analysis shows a normal Dynamic SPECT/CT but positive PCI group (5 patients), and less than normal MPI but positive PCI group (15 patients). 

### 3.5. The Evaluation of Ischemic Extent, Moderate Abnormal Perfusion, or Combined Ischemic with Moderate Abnormal Perfusion of LV Myocardium in Dynamic SPECT/CT According to PCI Results 

**Figure 2:**
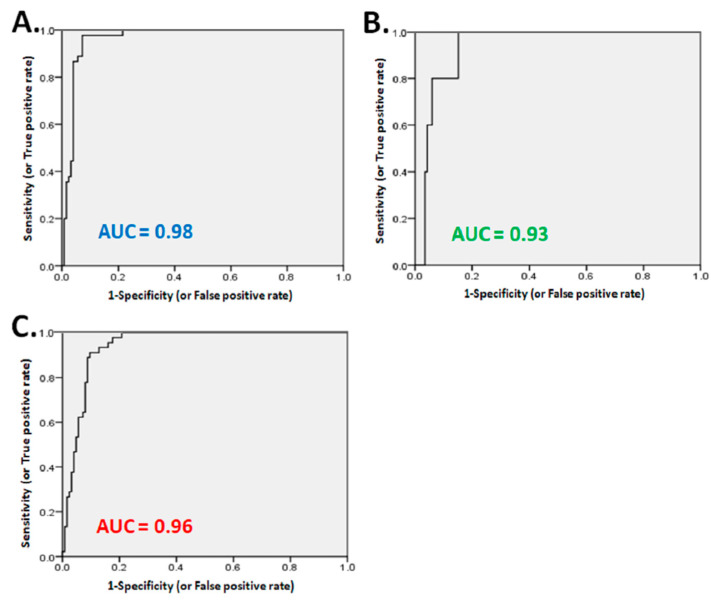
The evaluation of abnormal perfusion extent of LV myocardium in Dynamic SPECT/CT for predicting significant stenosis in patients. The area under the curve was 0.98(95% CI, 0.95–1.01), and if >5% of ischemic extent of LV that yielded significant stenosis (**A**), the sensitivity and specificity were 89.8% and 99.1%, respectively. The area under the curve was 0.93(95% CI, 0.83–1.02), and if >15.3% of moderate abnormal perfusion extent of LV that yielded significant stenosis (**B**), the sensitivity and specificity were 64.8% and 91%, respectively. The area under the curve was 0.96(95% CI, 0.90–1.01), and if >27.3% of combined ischemic and moderate abnormal perfusion extent of LV that yielded significant stenosis (**C**), the sensitivity and specificity were 77.8% and 97.3%, respectively. (AUC: area under curve).

The highest accuracy (96%) was acquired with >5% (cut-off extent: 5%) of ischemic extent in LV myocardium by the ROC curve which will predict significant stenosis during PCI intervention. The area under the curve (AUC) was 0.98 (95% CI, 0.95–1.01) (Figure 2A). The sensitivity and specificity were 89.8% and 99.1%, respectively. The accuracy (82.4%) was acquired with >15.3% (cut-off extent: 15.3%) of moderate abnormal perfusion extent in LV myocardium by ROC curve analysis. The area under the curve (AUC) was 0.93 (95% CI, 0.83–1.02) (Figure 2B). It shows sensitivity and specificity with 64.8% and 91%, respectively. For the combined ischemic extent with moderate abnormal perfusion extent evaluation, the accuracy (90.9%) was acquired with >27.3% (cut-off extent: 27.3%) of combined ischemic and moderate abnormal perfusion extent of LV by ROC curve which yielded significant stenosis. The area under the curve (AUC) was 0.96 (95% CI, 0.90–1.01) (Figure 2C). The sensitivity and specificity of it by PCI was 77.8% and 97.3%, respectively.

## 4. Discussion

Our prospective study enrolled patients with significant suspicion of CAD who were referred to our department by cardiologists for Dynamic SPECT/CT evaluation. After we reviewed articles, there are very few studies discussing the percentage extent of ischemic myocardium on Dynamic SPECT/CT to predict significant stenosis. In this study, we not only head-to-head compared semi-quantitative MPI and quantitative Dynamic SPECT/CT results, but also evaluated the severity of perfusion extent of LV myocardium in Dynamic SPECT/CT according to post PCI results. We also concluded the cut-off extent of abnormal perfusion of patients with significant suspicion of CAD who should be considered for revascularization. According to our result, we suggested overall monitoring of semi-quantitative MPI, MBF, CFR, ischemic extent, and moderate abnormal perfusion extent of Dynamic SPECT/CT before revascularization for optimal treatment of patients with significant suspicion of CAD. In our study, sensitivity (64.3%) and specificity (79.2%) of MPI was noted which presented moderate diagnostic value. However, we identified 26 patients with positive MPI results, but normal results of PCI. Reviewing PCI results in our patients, there are some patients with 50–70% coronary stenosis with normal MPI results. We defined >70% coronary stenosis to be significant stenosis in this study. Some patients had positive MPI results but with 50–70% coronary arteries (nonsignificant stenosis) and may yield this result. 

^99m^Tc-MIBI was used to be the cardiac tracer in this study. We found some patients had hyperactivity in the inferior wall of LV myocardium. Patients were told to be fasting at least eight hours before examination, in order to have an empty stomach and increased liver activity during the MIBI intravenous injection. The inferior wall hyperactivity may cause MPI imaging analysis interference caused by overlapping of the left liver and inferior wall myocardium, which especially underestimates the LAD perfusion in semi-quantitative analysis. The discordance of underestimated LAD perfusion in MPI, but preserved coronary flow in Dynamic SPECT/CT, was noted in some patients. Dynamic SPECT/CT shows quantitative coronary blood flow analysis with CT attenuation correction for soft tissue in the nearby myocardium. However, we did not use CT attenuation for imaging analysis in MPI. Due to this, the hyperactivity of the inferior wall might lead to underestimation of left anterior descending (LAD) perfusion after MPI imaging analysis. The light meal intake may be suggested before study to avoid overlapping of left liver and inferior wall myocardium, which is more favored in semi-quantitative analysis. 

In previous studies, conventional myocardial perfusion SPECT is a well-known issue and plays an important role for diagnosis of CAD, including prediction of the prognostic value to patients by noninvasive imaging modality [19,20,21]. Although MPI has higher sensitivity, there are still some present limitations [22], causing triple vessel disease, myocardial infarction, cardiomyopathy, or body attenuation. Cardiac PET with myocardial perfusion tracers with ^13^N-Ammonia (NH3) or ^82^Rb for quantitative MBF and CFR of coronary artery has been also considered as a useful tool for diagnosis and prognosis of CAD [23,24,25]. However, it sometimes presents limitations due to expensive costs or cyclotron availability in Taiwan. Hsu and coworkers presented quantitative MBF and CFR evaluation by Dynamic SPECT/CT to enhance CAD detection [10], similar to PET/CT. After reviewing prior studies, Gould et al. reported the CFC region with a specific artery distribution of quantitative cardiac PET/CT associated with major adverse cardiovascular events (MACE) [18]. It showed reduced CFC with CFR ≤1.27 and stress perfusion ≤0.83 cc/min/g that increased the hazard ratio for MACE and also all causes of death, MI, and stroke. Another report revealed severely reduced CFR (1.0 to 1.5) and stress perfusion ≤1.0 cc/min/g, leading to highest MACE and reduced by revascularization [26]. The global values are stress blood flow 1.8 cc/min/g and CFR 2.7. In our study, the mean values of stress blood flow 2.2 ± 0.8 cc/min/g, and CFR 2.6 ± 0.8 were noted in the normal group, stress blood flow 1.3 ± 0.3 cc/min/g, and CFR of 1.5 ± 0.4 in CAD group. It seems to be overestimated in MBF and CFR in the Dynamic SPECT/CT of this study, as compared with prior PET/CT studies. Quantitative coronary blood flow can also be obtained by the cardiac Cadmium-Zinc-Telluride (CZT)-SPECT camera. Agostini et al. presented that CZT-SPECT yielded higher stress and rest MBF, as compared with three procedures, but not in RCA territory. Stress and rest values are noted with: ^15^O-water PET in global LV (stress 3.18±0.95 mL/min/g; rest 1.15±0.31 mL/min/g; CFR 2.82±0.96), LAD (stress 3.25±0.97 mL/min/g; rest 1.25±0.32 mL/min/g; CFR 2.67±0.77), and LCx territories (stress 3.33±0.99 mL/min/g; rest 1.26±0.42 mL/min/g; 2.8±0.79) [27]. They also concluded a 2.1 cut-off value for CFR which predicted an abnormal fractional flow reserve (FFR) (<0.80) comparable to PET. The difference in the regional MBF and CFR values between CZT-SPECT and PET/CT was mentioned for the use of an attenuation correction on PET/CT. There is a noted mean value of rest MBF 1.04 ± 0.2 cc/min/g in the normal group, but no significant difference noted in the CAD group 1.1 ± 0.2 cc/min/g. Similar values of rest MBF are compared to rest flow of ^15^O-water PET in Agostini’s study. 

A SPECT-defined cut-off of approximately 10–15% ischemic myocardium was associated with equipoise between early PCI and medical therapy among patients without known CAD, and increasing survival after revascularization [28]. We defined >8% ischemic myocardium of LV on Dynamic SPECT/CT to be positive results for evaluation according to prior PET results. However, conventional MPI or cardiac PET/CT cannot be 100% reliable data for Dynamic SPECT/CT usage or further evaluation. Revascularization is not only increased MBF and CFR in CAD patients, but also decreased event rates and mortality have been noted in prior articles [5,23]. A recent meta-analysis study revealed that with suspected or known CAD patients, an impaired MFR is also associated with adverse cardiovascular events [29]. However, if there is a very small ischemic extent in LV, is it beneficial to undergo PCI treatment or medical treatment? Will this find be significant coronary stenosis for proper treatment before PCI? Our results concluded and presented the cut-off value of severity for abnormal LV by post-PCI results. Hopefully, it can be a useful tool for physicians before consideration of PCI treatment. Interestingly, moderate abnormal perfusion of our patients was considered for PCI intervention, if there was more than 15.2% abnormal perfusion. Moderate reduced coronary flows can be considered as ischemia-related flow range and can be associated with manifestations of ischemia in prior cardiac studies [6]. We identified significant moderate abnormal perfusion extent of LV by Dynamic SPECT/CT for prediction of significant stenosis of PCI. 

The present study had some limitations. First, our patients had no FFR data for comparison with Dynamic SPECT/CT. FFR is used to determine physiological significance of coronary lesions or stenosis, based on the pressure differential across the stenosis. We collected and defined one or more coronary arteries with a diameter of more than 70% stenosis by PCI. There is a lack of information between MBF, MFR FFR, or coronary stenosis. Second, our patients were referred from cardiologists due to significant suspicion of CAD, and who presented typical cardiac symptoms and might be considered for PCI. The results may have been influenced by a small sample size and selection bias. Third, cut-off optimized endeavors (training set equals evaluation set) not only naturally lead to overoptimistic results, but also no form of validation has been performed, not even cross-validation. 

## 5. Conclusions

Dynamic SPECT/CT not only provides quantitative MBF and CFR information, but also could be a valuable evaluation for pre-catheterization based on the abnormal extent of myocardium. This procedure has improved more than 10% sensitivity and specificity than those of semi-quantitative MPI, although it is overestimated in MBF and CFR, as compared with PET/CT published studies. This study concluded that the severity of an abnormal perfusion extent in LV might be used to predict significant coronary stenosis in patients with significant suspicion of CAD. Further prospective studies with larger numbers of cases are warranted.

## Figures and Tables

**Table 1:** Characteristics of the 181 enrolled patients in this study.

Age (Year) (Mean ± SD)	68.5 ± 7.2
Gender	
Male	99
Female	82
Smoking (Y/N)	32/149 (19%/81%)
Alcohol consumption (Y/N)	10/171 (1%/99%)
Caffeine consumption (Y/N)	72/109 (40%/60%)
Tea consumption (Y/N)	56/125 (31%/69%)
HTN(Y/N)	154/27 (85%/15%)
DM(Y/N)	113/68 (62%/38%)
Hyperlipidemia (Y/N)	91/90 (50%/50%)

HTN, hypertension; DM, diabetes.

**Table 2:** The relationship of MPI and cardiac catheterization in patients of this study.

		MIBI MPI		Total (N)
		No Ischemia	Had Ischemia	
Cardiac Catheterization	Normal	99	26	125
	Abnormal	15	27	42
Total (N)		114	53	167

N, patient number; MPI; myocardial perfusion imaging.

**Table 3:** The relationship of Dynamic SPECT/CT and cardiac catheterization in patients of this study.

		Dynamic SPECT/CT (Ischemic Extent of LV)		Total (N)
		<8%	≥8%	
Cardiac Catheterization	Normal	120	5	125
	Abnormal	5	37	42
Total (N)		125	42	167

LV, left ventricle; N, patient number.

**Table 4:** Comparison of MBF and CFR between normal patients and CAD patients.

	Normal Group (*n* = 125)	CAD Group (*n* = 42)	*p* Value
LV-CFR	2.6 ± 0.8	1.5 ± 0.4	<0.01
LV-stress flow (mL/min/g)	2.2 ± 0.8	1.3 ± 0.3	<0.01
LV-rest flow (mL/min/g)	1.04 ± 0.2	1.1 ± 0.2	0.058

CFR, coronary flow reserve; LV, left ventricle.

## Data Availability

The data presented in this study are available on request from the corresponding author.
